# Molecular phylogeny of grunts (Teleostei, Haemulidae), with an emphasis on the ecology, evolution, and speciation history of New World species

**DOI:** 10.1186/1471-2148-12-57

**Published:** 2012-04-26

**Authors:** José Julián Tavera, Arturo Acero P, Eduardo F Balart, Giacomo Bernardi

**Affiliations:** 1 Centro de Investigaciones Biológicas del Noroeste, S.C., La Paz,, B.C.S., México, USA; 2 Department of Ecology and Evolutionary Biology, University of California Santa Cruz, CA, 95060, USA; 3 Universidad Nacional de Colombia sede Caribe, CECIMAR/INVEMAR, Santa Marta, Colombia

## Abstract

**Background:**

The fish family Haemulidae is divided in two subfamilies, Haemulinae and Plectorhynchinae (sweetlips), including approximately 17 genera and 145 species. The family has a broad geographic distribution that encompasses contrasting ecological habitats resulting in a unique potential for evolutionary hypotheses testing. In the present work we have examined the phylogenetic relationships of the family using selected representatives of additional Percomorpha based on Bayesian and Maximum likelihood methods by means of three mitochondrial genes. We also developed a phylogenetic hypothesis of the New World species based on five molecular markers (three mitochondrial and two nuclear) as a framework to evaluate the evolutionary history, the ecological diversification and speciation patterns of this group.

**Results:**

Mitochondrial genes and different reconstruction methods consistently recovered a monophyletic Haemulidae with the Sillaginidae as its sister clade (although with low support values). Previous studies proposed different relationships that were not recovered in this analysis. We also present a robust molecular phylogeny of Haemulinae based on the combined data of two nuclear and three mitochondrial genes. All topologies support the monophyly of both sub-families (Haemulinae, Plectorhinchinae). The genus *Pomadasys* was shown to be polyphyletic and *Haemulon*, *Anisotremus*, and *Plectorhinchus* were found to be paraphyletic. Four of seven presumed geminate pairs were indeed found to be sister species, however our data did not support a contemporaneous divergence. Analyses also revealed that differential use of habitat might have played an important role in the speciation dynamics of this group of fishes, in particular among New World species where extensive sample coverage was available.

**Conclusions:**

This study provides a new hypothesis for the sister clade of Hamulidae and a robust phylogeny of the latter. The presence of para- and polyphyletic genera underscores the need for a taxonomic reassessment within the family. A scarce sampling of the Old World *Pomadasys* species prevents us to definitively point to a New World origin of the sub-familiy Hamulinae, however our data suggest that this is likely to be the case. This study also illustrates how life history habitat influences speciation and evolutionary trajectories.

## Background

Speciation rates of teleost fishes are likely to be influenced by a combination of abiotic (e.g. tropical and temperate environments) and biotic factors. Ecological factors that play an important role vary dramatically in coral and temperate reef habitats where overall number of species and their resulting biotic interactions are very different [1]. In addition, vast differences are found within the same habitat between regions, such as the Caribbean, where the fish fauna is relatively depauperate, and the Indo-Pacific, or the coral triangle, where marine fish diversity peaks [2,3].

Evolutionary history and speciation dynamics are difficult to compare directly between habitats and geographic regions because few groups of marine fishes span these entities. Haemulids, however, provide such an opportunity and are therefore a choice system to evaluate the mechanisms that are responsible for generating differences between habitats and regions. In addition, the presence of some putative geminate pairs of New World grunts [4] allows for an internal hypothesis testing of temporal patterns of speciation in this group of fishes.

Haemulidae is one of the ten most diverse, widespread, and conspicuous families within the largest sub-order of teleost fishes, the Percoidei [5]. They are commonly called grunts, due to their ability to produce loud sounds by rubbing their pharyngeal teeth together [6]. Haemulids tend to congregate during the day and then spread out for foraging at night. The family contains about 145 extant species currently classified in 17 nominal genera [5], grouped, based on morphological data, in two sub-families (Haemulinae, Plectorhynchinae) [7]. These two groups differ greatly in diversity and distribution. Haemulines, include most of the genera and are primarily distributed in the New World with the exception of *Pomadasys* (a genus that includes an estimated 36 species). Haemulines are diverse in shape (elongate or stout), ecology (different feeding modes and prey items), and habitat (temperate reefs, coral reefs, sandy and muddy bottoms) [8-12]. In general, haemulin grunts tend to be greyish and drab, with some exceptions such as the Caribbean porkfish, *Anisotremus virginicus,* which is mostly bright yellow with a few conspicuous black bars *.* On the other hand, sweetlips (Plectorhynchinae), are mostly restricted to the coral and rocky reefs of the Indo-Pacific and eastern Atlantic, and include approximately 50 species [7]. They are morphologically uniform, with an elongated body, a round head, and a subterminal mouth [13-15]. Sweetlips, colouring and patterning changes dramatically throughout their lives, and tend to be very distinctive, especially when compared to their haemulin counterparts.

**Figure 1:**
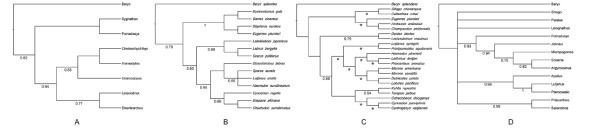
**Alternative phylogenetic hypotheses between Haemulidae and other percomorph families.A.** Bayesian tree of Dettai and Lecointre, 2005; **B.** Bayesian tree of Chen et al., 2007; **C.** Parsimony tree of Smith and Craig, 2007; **D.** Parsimony super-tree of Li et al., 2009.

Systematic and evolutionary history of haemulids has received little attention. The relationship between Haemulidae and other families, as well as its placement among percomorph fishes have varied through time according to different authors (Figure 1). Several attempts to include broad percomorph taxon sampling were done early on [16,17], however few of the characters found among percomorphs and their relatives were confirmed as uniquely derived [18].

Orrell and Carpenter [19] in their study of the phylogeny of Porgies (Sparidae) included Haemulidae, and some other percomorph families as outgroups. A parsimony reconstruction based on two mitochondrial genes (16 S and CYB), recovered the branch (Haemulidae (Lutjanidae-Caesionidae)) as sister to the sparids, however under maximum likelihood, the position of Haemulidae changed, placing it as sister to moronids and lutjanids. Caesionids and sparids clustered together as the sister group.

One of the first molecular studies that included haemulid species, with the particular intention of broadly examining percomorph relationships, was that of Dettai and Lecointre [20]. They found a weakly supported branch including *Pomadasys* and *Syngnathus* sister to (( *Lateolabrax-Dicentrarchus*) ( *Uranoscopus* ( *Ammodytes-Cheimarrichthys*))).

Chen et al. [21] recovered a polytomy of Lutjanidae, Scaridae, and Haemulidae using four different gene regions. Smith and Craig [22] used five genes fragments (4036 bp) with a broad panel of perciform fishes and recovered a monophyletic group that included the families Lutjanidae, Haemulidae, Lethrinidae, Priacanthidae, Moronidae and Lobotidae. Li et al. [23] used the gene RNF213 as well as a combined matrix of three different nuclear genes (MLL, Rhodopsin, IRBP), to build a supertree where Haemulidae appeared sister to Sciaenidae, a relationship that was not found in any previous molecular study [21,22].

Haemulidae and Inermiidae were included by Johnson [7] in the superfamily Haemuloidea. The bonnetmouths (Inermiidae) are a very small family of fishes with only two known species in two genera (*Inermia* and *Emmelichthyops*) [5]. These two species share derived highly protrusible jaws and elongated oval body conspicuously different from Haemulidae [7]. Rocha et al. [24] found *Inermia* nested within *Haemulon*. This result challenges the taxonomic status of *Emmelichthyops atlanticus*, it was suggested that this species may be placed provisionally in the family Emmelichthyidae (Heemstra and Randall, 1977), and Inermiidae should no longer be considered valid [24]. However Emmelichthyidae is an extant valid family [25] not closely related to *Emmelichthyops* despite its sharing the same etymological root, hence assigning *Emmelichthyops* to Emmelichthyidae is not a proper option.

The genus *Hapalogenys,* currently placed in its own family Hapalogenyidae (sic. Haplogeniidae) [26], has been commonly placed in Haemulidae [5,27,28], although Johnson [29] included it as “incertae sedis” within the Percoidei, as he found similarities with other non-haemulid groups. Different affinities on larval characters with *Lobotes* and *Datnioides* allowed Leis and Carson-Ewart [30] to place *Hapalogenys* in a group they informally called Lobotes-like, suggesting a possible relationship of *Hapalogenys* to lobotids. Clearly, more effort is needed to clarify the familial position of *Hapalogenys,* even though some authors have decided to leave it within Haemulidae in spite of being aware of strong dissimilarities [5,27,28].

Haemulidae relationships are controversial and have received little attention. A reassessment of the phylogenetic relationships of the family Haemulidae is therefore timely since neither morphological nor molecular studies have produced consistent results [5,7,16,17,20-22]. Our analysis includes representatives from lineages that have been found related to haemulids, however we did not include those families that have been inconsistently recovered as their relatives, although we included Sillaginidae following the advice of a colleague (R. Betancur, pers. comm). One of the objectives of this study was to use molecular data to test the monophyly and evaluate the intra- and inter-relationships (i.e., sister groups) of Haemulidae.

Within Haemulidae, molecular studies have attempted to elucidate relationships of two closely related genera, *Haemulon*[24] and *Anisotremus*[31]. Recent morphological studies [32] suggests that the latter is not monophyletic. Indeed, *Anisotremus* seems to be an assemblage of at least three different lineages with two previously recognized *Anisotremus* ( *A. dovii* and *A. pacifici*) being reassigned to *Genyatremus*. The monophyly of *Haemulon* was also shown to depend on the inclusion of *Inermia vitatta*. In summary, despite the sparse efforts to resolve the taxonomy of the Haemulidae, its generic nomenclature is still unstable.

This study provides the most inclusive phylogeny of Haemulidae and New World grunts using molecular data. These data were utilized for hypothesize haemulid intra and inter relationships discuss current classifications in the light of molecular phylogenies; and to explore the ecology, evolution, and speciation history of New World haemulids. These data allow to assess the monophyletic status of the two subfamilies, Haemulinae and Plectorhynchinae, determine their relationship, and set the groundwork to explore the relative roles of biotic and abiotic factors in the history of diversification that occurred in this group.

## Results

### Dataset structure

Two independent data sets were assembled: all mitochondrial genes together, and a combined-matrix of mtDNA, and nucDNA. The final mitochondrial alignment was 1789 bp long, with 806 variable sites. The combined-data consisted of 2909 aligned base pairs (bp), of which 1228 were variable. Detailed data set attributes are summarized on Table 1.

**Table 1:** Detailed information of datasets used

**Type of locus**	**Locus**	**Length**	**N mtDNAdataset**	**BF Model mtDNA dataset**	**PIC mtDNA dataset**	**N combined dataset**	**BF Model combined dataset**	**PIC combined dataset**
Mitochondrial	16 S	549	46	TIM2 + I + Γ	183	54	TIM2 + I + Γ	130
genes	COI	517	46	TIM2 + I + Γ	203	54	TIM1 + I + Γ	188
	CytB	723	46	TPM3uf + I + G	318	54	TIM2 + I + Γ	290
Nuclear exon	RAG2	662				54	TPM2 + Γ	117
Nuclear intron	S7					54	HKY + Γ	247

N stands for number of taxa; BF model, Best fit model; and PIC, Parsimony informative characters.

### Phylogenetic analysis

Maximum Likelihood trees obtained from independent runs in both RAxML and GARLI were identical, differing only in support values for which just one topology is presented. Different partition scenarios were separately analyzed and widespread congruence between them was found.

Mitochondrial genes share the same evolutionary history, its inheritance is clonal, which means that the whole genome behaves as a single, non- recombining locus [33]. This considerably simplifies their analysis as they have a common genealogy changing only individual gene patterns of evolution.

**Figure 2:**
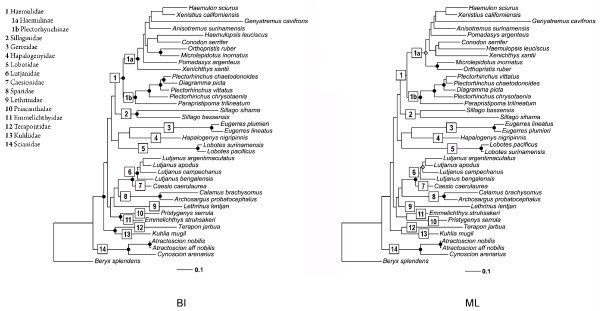
**BI and ML phylogenies between Haemulidae and other percomorph families derived from the mitochondrial dataset (1789 bp).** Left Bayesian inference tree ( **BI**): circles on nodes indicate posterior probability (pp) values: black circles (pp ≥ 0.95). Right Maximum likelihood ( **ML**): circles indicate support from five independent maximum likelihood bootstrap (bs) analyses, black circles (bs P ≥ 0.95), gray circles (0.70 ≤ bs < 0.95).

Mitochondrial phylogenetic information resulted in a newly uncovered relationship with the Sillaginidae sister to the Haemulidae, and both sisters to the branch ((Gerreidae-Hapalogenyidae)Lobotidae). However, the relationships among these percomorph taxa were poorly supported (Figure 2).

A different branch was found, where Emmelichthyidae was related to Priacanthidae with high nodal support and both together were recovered as sister to (Lethrinidae (Sparidae (Lutjanidae-Caesionidae))). Lutjanidae was highly supported as sister to Caesionidae. Terapontidae and Kuhlidae grouped together with a high nodal support and are positioned as the sister-species to all the previously mentioned families. Finally Sciaenidae appears as the basal branch among the families included in this study (Figure 2).

The putative sister relationship between the two subfamilies Haemulinae (New World grunts) and Plectorhynchinae (sweetlips) was recovered by all analyses (i.e. partition schemes and exploratory methods) with high support values (Figure 2). A relatively deep divergence (from 24 up to 41% corrected genetic distance across all the combined genes) existed between these two clades. Corrected genetic distances within Haemulinae genera ranged from 1 to 34% whereas in Plectorhynchinae they ranged from 8 to 15%. However, for sweetlips, these values were likely to be underestimated due to the small number of species included. Indeed, while sampling was comprehensive for the haemulines, including all but some local endemic species, the amount of sweetlips species sampled was not an extensive representation of their diversity. However, and in spite of the few species included, the genus *Plectorhinchus* was found to be paraphyletic by the nested inclusion of *Diagramma picta* (Figure 2). The type species of the genera *Plectorhinchus* ( *P. chaetodonoides*) and *Diagramma* ( *D. picta*), were included in our study and were found to be sister species, with their clade embedded within *Plectorhinchus*, and closely related to *P. vittatus* with a high nodal support *.* Our data placed *Parapristipoma trilineatum* as the sister species to the remaining *Plectorhinchus* used in this study (Figure 2).

**Figure 3:**
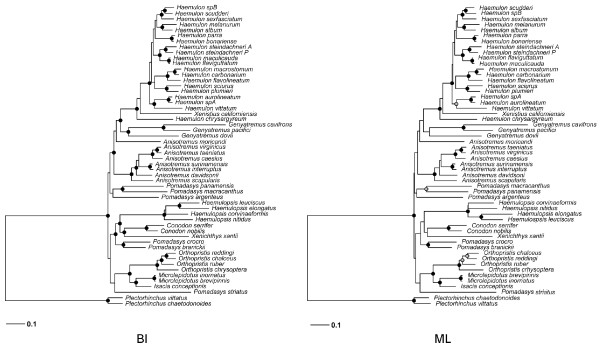
**BI and ML phylogenies among Haemulinae species derived from the combined (mitochondrial and nuclear) dataset (2909 bp).** Left tree corresponds to BI tree and circles on nodes indicate posterior probability (pp) values: black circles (pp ≥ 0.95). Right tree is the ML tree. Circles indicate support from five independent maximum likelihood bootstrap (bs) analyses: black circles (bs P ≥ 0.95), gray circles (0.70 ≤ bs < 0.95).

**Figure 4:**
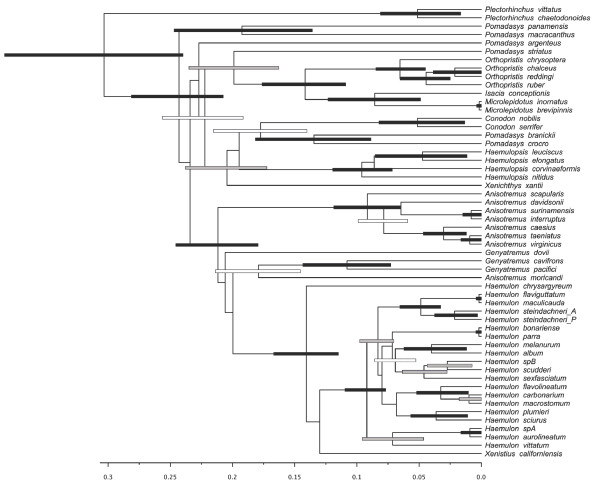
BI time–relative tree from *BEAST derived from the combined dataset. Geminate species inhabiting basin is indicated with (WA) for western Atlantic while (EP) stands for eastern Pacific. 95% HPD node bars are filled according to posterior probability. Black bars (pp ≥ 0.95), gray bars (0.70 ≤ pp < 0.95), white bars (pp < 70%).

**Figure 5:**
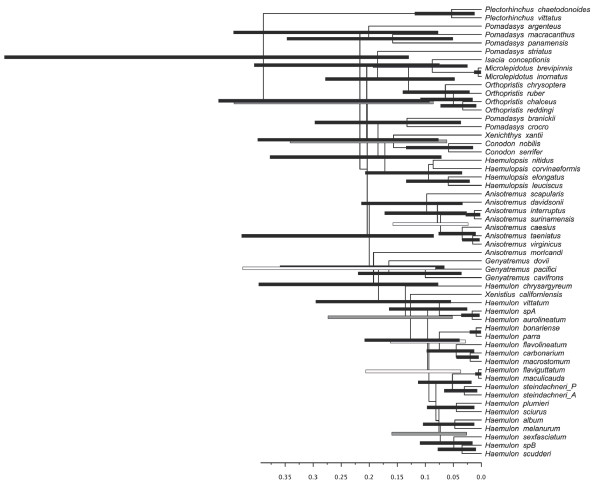
**BI time–relative tree from the concatenated genes matrix under BEAST.** Geminate species inhabiting basin is indicated with (WA) for western Atlantic while (EP) stands for eastern Pacific. 95% HPD node bars are filled according to posterior probability. Black bars (pp ≥ 0.95), gray bars (0.70 ≤ pp < 0.95), white bars (pp < 70%).

Within Haemulinae, all the topologies obtained with different methods were largely consistent with the existence of several clades with high nodal support throughout (BI ≥ 0.95; RAxML ≥ 95%; Garli ≥ 95%). However, relationships among some of them were not well resolved (Figure 3,4 and 5). Comparing p-values of the SH test, the mean estimate from the five genes combined matrix falls within the confidence limit of each individual gene and partition scheme, even though the individual estimates differ. This can be attributed to stochastic variation, and the combined matrix can be accepted as a good estimate of the parameters [34].

*BEAST topology (Figure 4) was highly similar to that of the BEAST supermatrix (i.e., concatenated) (Figure 5), however the position of *Anisotremus moricandi, Genyatremus dovii, Xenichthys xantii,* and that of the branch including *Pomadasys argenteus*, *P. macracanthus* and *P. panamensis* differed among these methods.

Twelve genera were sampled, eight of them comprising more than one described species. Out of those eight, the monophyletic status of five genera, *Conodon, Haemulopsis, Genyatremus, Microlepidotus,* and *Orthopristis* was consistent with our data. In contrast, the monophyly of the largest New World genus, *Haemulon*, was challenged by the inclusion of one species. The second largest New World genus, *Anisotremus*, was composed of a monophyletic assemblage of seven species. In the concatenated methods the Southern Caribbean and Brazilian brownstriped grunt, *Anisotremus moricandi,* did not cluster with any other species, but was closely related to the main *Anisotremus* clade. However the *BEAST search placed it as sister to *Genyatremus pacifici* and *G. cavifrons*, yet with a very low posterior probability. The former two *Anisotremus* species, now *G. dovii* and *G. pacifici* grouped together with the previously monotypic *Genyatremus* in the supermatrix analyses *.* These results are consistent with those presented by Tavera [32] and Bernardi [31]. Nevertheless in multilocus coalescent analysis, *G. dovii* was recovered as the sister species to the branch including remaining *Genyatremus* +  *Anisotremus moricandi* and the clade ( *Haemulon* +  *Xenistius*).

The largest genus of the subfamily, *Pomadasys*, which contains an estimated 36 species with a very wide distribution, was found to be paraphyletic *,* with species being widespread in the tree in three different branches. ML reconstructions forcing *Pomadasys* into a monophyletic group resulted in topologies that were significantly worse than those obtained under unconstrained searches (SH test p = 0.00).

The presence of new species, and the polyphyly and paraphyly of several genera underscores the necessity for a thorough systematic revision of the family at this stage.The subfamiliy Haemulinae was found to be monophyletic, and a single origin for New World grunts was recovered, but the inclusion of two Old World species, *Pomadasys argenteus* and *P. striatus* (Figure 3‐5), suggests that not all species were retained in this geographical area, yet the position of these species within the New World clade was not strongly supported. An extensive sampling of Old World “*Pomadasys*” would be needed to properly address this issue.

Haemulinae saturation analyses revealed some saturation at the third codon position in the mitochondrial gene CYB at large genetic distances (comparisons between ingroup and outgroup) but not within the ingroup. No saturation was observed for other genes in any position.

### Time of evolutionary divergence

Evolutionary divergence times in Haemulinae was evaluated using four trans-Isthmian geminate pairs (see [35], for a review), *Anisotremus interruptus – A. surinamensis, Anisotremus taeniatus – A. virginicus, Conodon nobilis – C. serrifer,* and *Haemulon steindachneri* Pacific *– H. steindachneri* Atlantic. *Pomadasys branickii – P. crocro* were also described as a geminate pair [4], and although, our data place them taxa as sister species, the absence of samples from *P. bayanus,* a species that is morphologically and ecologically similar to *P.crocro*, precludes us from conclude if *P. branickii* and *P. crocro* are true geminates. The species pairs, *Haemulon scudderi – H. parra and H. sexfasciatum – H. album,* were also considered as trans-Isthmian sisters [4,12] but morphological similarities in these pairs were found to be morphological convergence rather than common ancestry. All analyses consistently found reciprocal monophyly for the four *bona fide* geminate pairs, but the magnitude of genetic divergence (i.e. corrected genetic distance) among them is inconsistent with simultaneous isolation, in spite of the overlapping of high credibility intervals in the nodes, as shown in Figure 4‐5.

**Figure 6:**
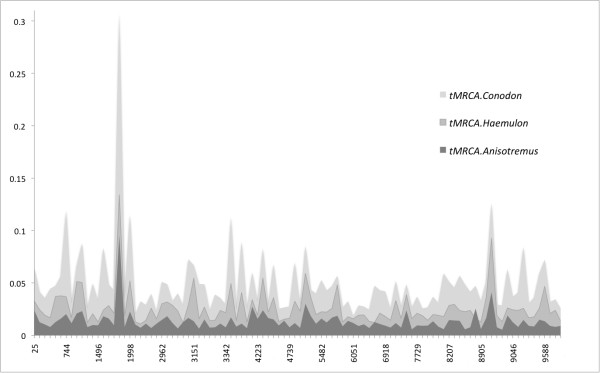
100 random samples of tMCRA from three geminate nodes taken from 10000 *BEAST posterior trees. Nodes used were ( *A.surinamensis* +  *A.interruptus*, *C.nobilis* +  *C.serrifer*, *H.steindachneri* eastern Pacific +  *H.steindachneri* western Atlantic).

These differences can be associated with two different hypotheses: i) an issue of differential substitution rate across lineages; or ii) a difference in time of divergence between geminate species, which in turn has three possible scenarios. Smaller divergence may be attributed to a secondary contact between two former species during the recent breaching of the Isthmus of Panama that occurred approximately 2 Mya [36], as opposed to 3.1-3.5 Mya the generally accepted time of the final closure of the Isthmus [37], or speciation in some pairs began before the definitive emergence of the Isthmus. Under a time relative run (BEAST), rates were found to be similar across all four pairs, favouring the second assumption, where divergence events are not occurring simultaneously in time. To test this hypothesis, every tMCRA value of all geminate pairs was extracted from the posterior density of trees and compared among them. In 69% of the trees the tMCRA of the sister pair *A. taeniatus + A.virginicus* was older than that of *A. interruptus + A.surinamensis* with a Ln Bayes factor of 0.839; while in 99-98% of the trees the tMCRA of *Haemulon* sister pair was older than any *Anisotremus* pair (Ln BF: 4.88-3.86); and in 98% *Conodon* tMCRA was older than *Hamulon* Ln BF = 3.87. Yet, CI tMCRA nodes overlap among all but *Anisotremus* pairs, differences among relative divergence times were identified as shown on Figure 6.

### Reconstruction of habitat and ancestral distribution areas

**Figure 7:**
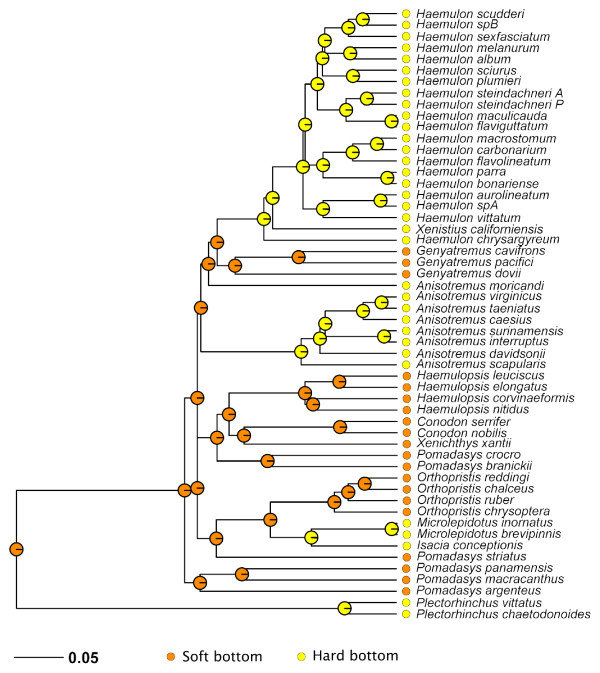
**ML reconstruction of habitat types mapped onto the BEAST tree.** ML ancestral reconstruction under all rate model (ARD). Habitat was coded accordingly to published references.

The ML reconstruction for ancestral habitats (soft bottom = character state 0=; hard bottom = state 1) in haemulinae is shown in Figure 7. This reconstruction was based on a discrete character for which two possible rate models were tested. ER, which implies one single rate and ARD that allows directionality. Model likelihoods were −15.74729 for ER and −13.79735 for ARD. ER and ARD models likelihood test yields a p-value of 0.0483 favouring the later; ARD includes more parameters that the ER, and it is well known that adding parameters to a model generally increases its likelihood.

New World grunts evolved over large geographic areas in two main habitats, soft bottoms (sandy and/or muddy) and hard bottoms (rocky and/or coral reefs). This ecological separation is highly concordant with recovered phylogenies of major lineages. The ancestral habitat of the most recent common ancestor for New World grunts is most likely soft bottom. The hard bottom habitat state occurs in *Haemulon*, *Anisotremus* (sensu lato) and *Microlepidotus.*, According to these results, haemulinae grunts shifted their habitats from soft to hard bottom, and not vice versa, and they independently invaded hard substrates from soft bottoms three separate times (Figure 7).

**Figure 8:**
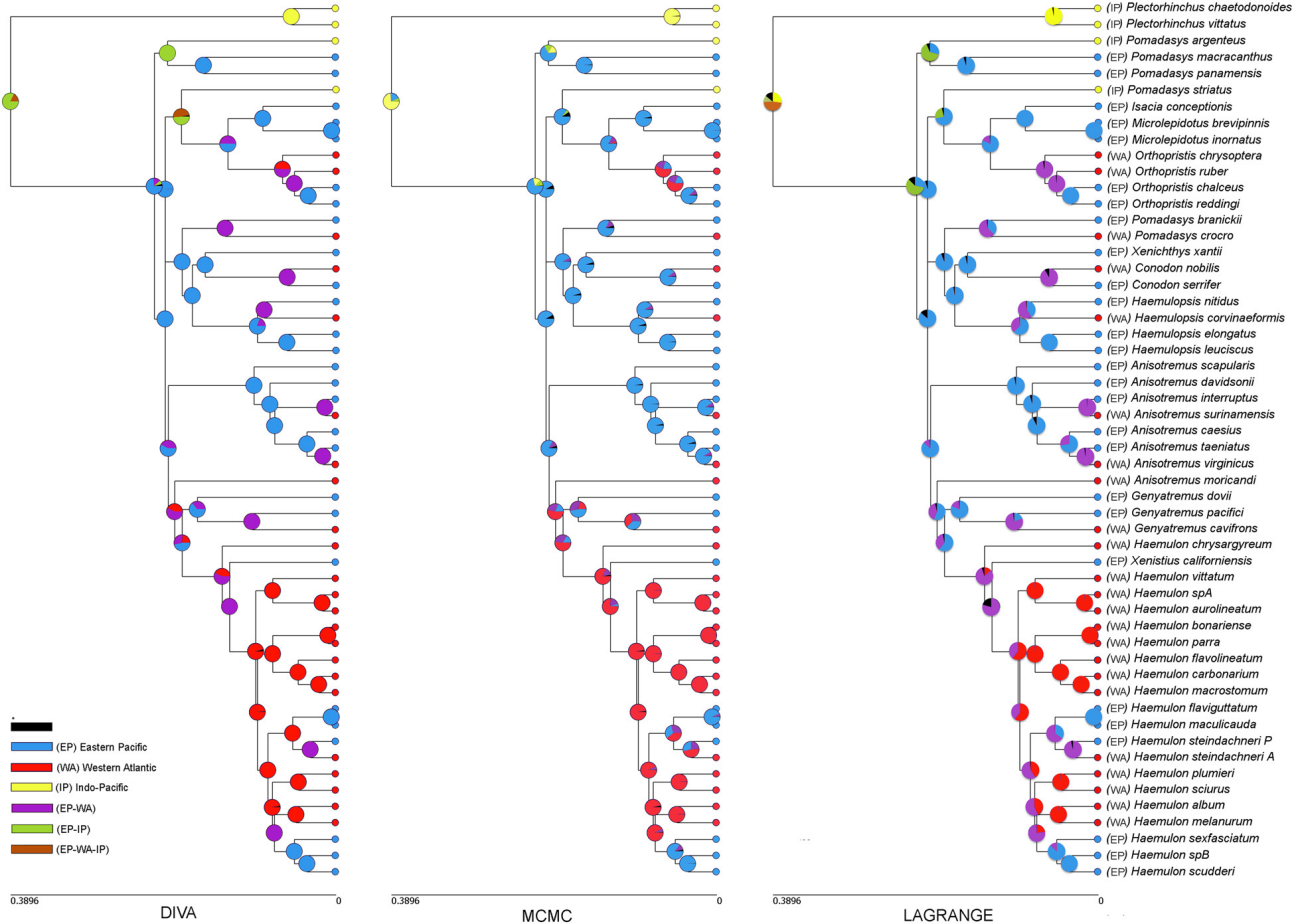
**Reconstruction of ancestral areas mapped onto the BEAST tree.** Three different reconstruction methods are depicted. Left was built based on S-DIVA. Center bayesian DIVA and right DEC under LAGRANGE.

The inferred historical biogeographic scenarios from analyses using LAGRANGE (DEC) and RASP (S-DIVA, and Bayesian) are presented in Figure 8. The inferred ancestral areas at internal nodes estimated using the Bayesian RASP correspond largely to the results obtained from the ML habitat reconstruction (Figure 7). RASP also estimated less combined ancestral areas than DEC and S-DIVA. The maximum likelihood reconstruction of ancestral areas for the basal node of haemulines differs from RASP reconstructions in that the most recent common ancestor (tmrca) of this clade most likely appeared in a broad area including the eastern Pacific and the Indo-Pacific (i.e., Pacific ocean), while in the other two methods the eastern Pacific was the most probable region from which this lineage could be originated. Range expansion into the western Atlantic occurred later, mainly with the MCRA of *HaemulonGenyatremus,* and *Anisotremus moricandi*. More recent transition events across eastern Pacific and western Atlantic occurred in New World grunts evolutionary history, when the final closure of the Panamian isthmus had not yet occurred. This geological event has been widely related to species divergence and its final closure estimated during the Mid Pliocene about 3.5-3.1 MyBP, however it has been demonstrated that the isthmus became an ecological barrier much earlier [38].

## Discussion

### Taxonomic remarks

Haemulids have been included among the so-called “lower percoids” and both molecular and morphological phylogenetic hypotheses have been proposed [7,19]‐[23,28]. Nevertheless haemulid familial relationships were never the main focus of any of these studies.

Our results differed from all previous works in placing the Sillaginidae as sister to Haemulidae (Figure 2). Sillaginidae and Haemulidae have been included in previous studies, as that of Smith and Craig [22] who recovered a branch including ((*Sillago-Callanthias*)( *Pseudupeneus-Dactylopterus*)) inside a basal group distant from Haemulidae. On the other hand, Li et al. [23] recovered *Sillago* as a single branch among a large politomy that also included Haemulidae + Sciaenidae.

Our study consistently recovered a branch comprising Lobotidae (Gerreidae-Hapalogenyidae) as the sister group to Haemulidae + Sillaginidae. A closer relationship between *Hapalogenys* and Haemulidae has been speculated upon phenotypic resemblance. However, Johnson [29] found morphological affinities with other groups (non haemulids), such as the rugosity on the frontal bone surface similar to a few apogonids, bramids, and serranids as well as to *AcanthocepolaLobotesPseudopentaceros* and *Sphyraenops,* and the shape of the spine-like crest that projects beyond the posterior margin of the cranium that is well-developed in preflexion larvae. This type of crest characterizes larval cepolids, leiognathids, lethrinids, (lobotids?), pentacerotids, priacanthids, and *Scombrops*. This piece of evidence along with previously published results [30] and our molecular data suggest a possible relationship between *Hapalogenys* and Lobotidae, in spite of low support (Figure 2).

Lutjanidae and Caesionidae were found as a clade with high support values, in both search methods, (Figure 2), which is consistent with the morphological Lutjanoidea [7]. These two families (Lutjanidae, Caesionidae) were found to be related to Sparidae and Lethrinidae, two of the four families proposed by Johnson [7] to be members of the super-family Sparoidea. This putative group was not recovered as a monophyletic assemblage by our data. Within this same branch and in a basal position, we recovered Priacanthidae and Emmelichthyidae as sister taxa with high support, for both search methods (i.e. Bayesian or Maximum Likelihood). This finding differs from Smith and Craig [22] who suggested a relationship between Priacanthidae and Lethrinidae. Not only did our data fail to recover them as sibling groups, but also placed them in very different positions along the tree (Figure 2).

Terapontidae and Kuhliidae were recovered together in a strong supported clade, sister to all other aforementioned families (Figure 2). This relationship is entirely consistent with data from Smith and Wheeler [39].

Finally Sciaenidae was recovered as a single branch, sister to all other groups included in this study (Figure 2), which disagrees with Li et al. [23] where sciaenids are sister to haemulids (Figure 1D). Our results differed from those of Chen et al.[21], Orrell and Carpenter [19], and Smith and Craig [21] (Figure 1), while partially concur with those presented by Rosen [17] who concluded that Johnson’s [7] Sparoidea and Hamuloidea were related to his vision of Pharyngognathi including Gerreidae (Figure 2).

This study is consistent with previous morphological division of haemulids into two major lineages, Plectorhynchinae (sweetlips) and Haemulinae [7]. Indeed, both groups were found to be reciprocally monophyletic for all five molecular markers used.

Within the sweetlips, *Diagramma picta* (Thunberg 1792) was nested inside *Plectorhinchus*. This finding suggests that *Diagramma* Oken 1817 may be considered synonym of *Plectorhinchus* Lacepède 1801, a possibility first raised by Konchina [40].

Within Haemulinae, the Bayesian coalescent search (i.e. *BEAST) recovered an early split of *Pomadasys macracanthus* and *P. panamensis* from the remaining species, followed by *P. argenteus* as a single branch, while the concatenated method (ie BEAST) places them in the same clade. Regardless of this topological difference, data suggest that these three species (possibly together with other unsampled Old world *Pomadasys* species) represent early basal haemulines.

The clade including the remaining haemulines was split in two different lineages, which match ecological differences. One group included species associated with soft bottom environments, and the other clade included mainly species inhabiting hard bottom (e.g. *Haemulon, Anisotremus*) (Figure 7).

Within the soft bottom clade, the circum-globally distributed genus *Pomadasys* has the largest number of species (36), but our results show it to be polyphyletic (Figure 3,4). Lacépède 1802 designated *Pomadasys argenteus* (Forsskål 1775) as the type species; therefore any species clustered together may be ascribed to *Pomadasys*. According to the coalescent tree method (i.e. *BEAST), *P. argenteus* did not cluster with any other Old or New World “ *Pomadasys*” species sampled. In contrast, standard concatenated method (i.e. BEAST) grouped together ( *P. argenteus* ( *P. macracanthus-P. panamensis*)) indicating that further efforts are needed to extensively sample this genus, mainly Old world species which were left out of this study. Still our data are sufficient to designate some new generic names in order to keep natural groups concordant with taxonomic rules, like, *P. crocro* and *P. branickii* clustered in a monophyletic assemblage. These species were originally described as *Pristipoma* Quoy and Gaimard (ex Cuvier) 1824. However, this genus name is not valid for any haemulid as its type species, *Pristipoma sexlineatum,* belongs to the family Terapontidae. On the other hand *Rhonciscus,* erected by Jordan and Evermann [41], as a subgenus under *Pomadasys* was already used to include these two species and is an available name, and thus eligible.

Conversely, some *Pomadasys* species may be included in pre-existing genera. For example, *Pomadasys corvinaeformis,* is recovered within the otherwise paraphyletic *Haemulopsis.* In fact, *P. corvinaeformis* was originally designated by Steindachner 1869 as the type species of *Haemulopsis*, an observation that is consistent with our own findings and the complex taxonomic history of the genus [42].

Our data recovered a well-supported clade that included *Anisotremus, Haemulon, Genyatremus,* and *Xenistius* (Figure 3‐4). Relative positions of each genus were only weakly supported in ML methods while strong posterior probabilities support *Genyatremus* as sister to *Haemulon + Xenistius + H.chrysargyreum*. On the other hand *A. moricandi* was recovered as an intermediate single branch, sister to these two lineages with 0.82 posterior probability, finally *Anisotremus* (sensu stricto) was found as the basal sister branch of this clade.

*Haemulon* is the most speciose taxa within the New World grunts including two undescribed, cryptic species (Figure 3). *Haemulon* was recovered as paraphyletic due to the inclusion of *Xenistius californiensis*, which clustered with *H. chrysargyreum* as deeply divergent basal branches. The relationship between these two species remains unclear as weak support was found in both methods as to preclude definitely conclusions, however topology indicates they are single units and additional data (i.e. morphological, more loci) are needed to address this issue, yet if they belong or not to *Haemulon* is a open question and depends on whether splitting or lumping philosophy is followed. Our data therefore suggest that either *Xenistius* is to be considered a *Haemulon*, or *H. chrysargyreum* be taken out of *Haemulon* (the genus *Brachygenys* would be available to this effect). The remaining *Haemulon* species are monophyletic with high support values.

The genus *Genyatremus* sensu Tavera et al. [32] was recovered as monophyletic by including two former *Anisotremus* species (Figure 3). In contrast, *Anisotremus* (sensu *lato*) was polyphyletic, with *A. moricandi* nested among other clades (i. e *Anisotremus, Haemulon, Genyatremus*). *Anisotremus moricandi* exhibits morphological characters of both *Haemulon* and *Anisotremus*, and has specific ecological requirements distinct from other tropical grunts [43] and also present a very unique biogeographical distribution, which make it a very interesting case among New World grunts and deserves further attention.

### Haemulinae evolutionary history

All analytical approaches, including the two different time-relative methods (BEAST and *BEAST) based on a relaxed molecular clock (Figure 4‐5), produced a well-resolved and congruent phylogeny of the 57 haemulid species. Concatenation methods (i.e. BEAST) assumes that all the data have evolved according to a single evolutionary tree, ignoring the occurrence of different evolutionary histories at different loci which in turn may result in well supported but incorrect species tree, while in coalescence methods (i.e.*BEAST) the inferred species tree is the one that minimizes the number of deep coalescences needed for the species tree to be compatible with each gene tree [44]‐[46]. Our results are consistent in recovering the same well-supported clades under both methods, with the exceptions treated above; however branch lengths, node heights, and HPD node intervals are substantially sensitive to the method used (Figure 4‐5).

In previous studies, different levels of genetic divergence have been observed for multiple trans-isthmian species pairs [35] such as snapping shrimp [47,48], bivalves [49], and now grunts (Figure 4‐5, Table 2). We positively identified at least three different stages for divergence among geminate species, in both concatenation and coalescence tree species method.

**Table 2:** Corrected genetic distances between grunts geminate species across the five genes used in this study

	**CYB**	**COI**	**16 S**	**S7**	**RAG2**	**Combined**
*A. surinamensis–A. interruptus*	0.02260	0.02188	0.00366	0.00453	0.00304	0.01282
*A. taeniatus–A. virginicus*	0.03159	0.01775	0.00550	0.001954	0.00610	0.01576
*C. nobilis–C. serrifer*	0.09830	0.10341	0.02423	0.001617	0.00608	0.05615
*H. steindachneri* EP *–H. steindachneri* WA	0.05509	0.07234	0.01480	0.003124	0.00151	0.03449

New World grunts present a unique opportunity to study the role of habitat, geographic origin, genetic divergence, and diversification times. Very early in the history of Haemulidae, sweetlips (Plectorhynchinae) diverged from other grunts (Haemulinae). In addition to genetic and morphological diversification, ecological diversification plays an important role in the evolutionary history of a lineage [50]. Sweetlips radiated in the Indo-Pacific into a group of approximately 50 species that are homogeneous in body shape and habitat (coral reefs) but differ greatly in color pattern, a feature that is typical of coral reef species where vision, in clear water, plays an important role in predator avoidance and mate recognition [51]. During the same period of time, haemuline grunts diversified into more than 110 species by invading a wide array of regions (Pacific and Atlantic oceans) and habitats (temperate and coral reefs, muddy and sandy bottoms).

ML ancestral reconstruction of haemuline recovered three independent hard bottom-invading events (Figure 7). Accordingly, the ancestral lineage was a soft bottom inhabitant distributed somewhere on the Pacific Ocean (Figure 8). This ancestor would have been distributed in habitats much like those existing throughout much of the eastern Pacific today. With respect to ancestral areas, although inferences differed in respect to some details, the methods converged upon a Pacific origin of the group, with a later dispersion into the western Atlantic with some recent vicariant events (e.g., rise of Panamian isthmus). However, our approach does not allow a precise determination of the origin of the New World grunts’ most recent common ancestor, perhaps an extensive sampling of Old World *Pomadasys* would shed light on this topic. Additional time node calibrations will also further our understanding of the timing of the Haemulidae evolution.

Geography and habitat association may have had a synergistic impact in shaping haemuline diversity. Indeed, the two basins (western Atlantic and eastern Pacific) were a single unit during most of the time of diversification of this group, and there is no evidence of limited dispersal capability in this family. Moreover, as shown by the genus *Pomadasys* (sensu *lato*), where Indo-Pacific species are nested within New World representatives, even broader geographic barriers have been breached several times during grunt evolution, but perhaps habitat and not geography affected grunts after invading western Atlantic with striking differences in substrate, which may in turn have caused a shift in rate diversification, as shown in Figures 6 and 7. In general, western Atlantic species live in relatively clear waters, as opposed to their eastern Pacific counterparts. Speciation in the two oceans followed different paths, more lineages but with fewer species emerged in the Pacific Ocean (Figure 8). In the eastern Pacific, water turbidity may have shaped speciation processes of the group. Indeed, while coral reef species are colorful, species that live in turbid water are not. Perhaps sound evolution may have had an unknown impact and need to be studied. Sound is used in mate recognition in several groups of fishes, including hamlets (*Hypoplectrus,* Serranidae) and in damselfishes (Pomacentridae) [52]. Similarly, grunts, which have the ability of producing sound, are good candidates for the study of mate recognition based on sound production.

Speciation in grunts might follow the stages of evolutionary radiation proposed by Streelman and Danley [53]. The first stage involves habitat utilization, followed by morphological specializations related to trophic resources and finally sensory communication.

Current geographic distribution of sister species exhibits a pattern perhaps related to habitat choice and shared evolutionary history. Whereas most *Haemulon* sister pairs are sympatric [24], sister species from *Microlepidotus* and *Orthopristis* have ranges that do not significantly overlap but are adjacent to each other, sometimes with species occurring together only in narrow zones. In the absence of geographic barriers this may suggest that parapatric speciation was the more likely mode of divergence for these clades. On the other hand, *Anisotremus, Conodon*, and *Genyatremus* exhibit an allopatric pattern where diversification could be related with different stages of the rise of the Panamian isthmus. This pattern is mirrored in gobies, where sympatric sister species are common in *Tigrigobius* and *Risor* and mostly absent in *Elacatinus*[54].

## Conclusions

This work strongly supports the monophyly of the previously proposed subfamilies Haemulinae and Plectorhynchinae. While our phylogenetic hypotheses are robust at the subfamily and generic level, some questions remain unsolved. The inclusion of additional samples will test the existence of monotypic lineages (e.g. *Xenocys, Brachideuterus,* and *Boridia*), and will allow exploring the relationships within sweetlips (i.e. Plectorhynchinae). However, we do not expect the general picture of the evolution of the grunts to be radically modified by the inclusion of such missing taxa.

Our work provides a framework to understand the factors that played a role in the diversification of the group. New World grunts were clustered in two major ecological groups, however hard bottom affinity seems to have independently evolved three times during haemuline history. Diversification events appear to be related with that ecological division, generating more lineages with less species in soft bottom habitants, while few but specious taxa populate the hard bottom environments, suggesting more specialization than previously suspected.

New World grunts may have originated in the Pacific Ocean with later dispersal into the western Atlantic with some recent reversal invasions, followed by vicariant events. The closure of the Isthmus of Panama, which resulted in allopatric divergence in a large array of marine organisms, played a role in grunts; yet geminate haemulids seem to have diverged at three different stages of this geological event.

## Methods

### Collection

Specimens were either collected at fish markets and fish camps, or directly by spear while scuba or skin diving. Some geographically widespread species were represented by up to four individuals from different collecting sites. All necessary permits were obtained for the described field studies accordance with University of California Santa Cruz’s Institutional Animal Care and Use (IACUC) Protocol # Berng1101.

Here we obtain new data from two nuclear loci and three mitochondrial genes for approximately 300 individuals corresponding to 69 species, including 60 grunts and 9 relatives, additionally the mitochondrial genes for 13 non-haemulid species were obtained from Genbank (Additional file 1). 50 of the 64 currently valid species of grunts in the New World and all but two of the thirteen genera were included (Table 3). All newly determined DNA sequences were deposited in Genbank (accession numbers JQ740898-JQ741944; see Additional file 1: Table S1 for details).

**Table 3:** Haemulidae species included in this study

	**Genus**	**Ocean basin**						**Sampled**	**Total**
		**New World (NW)**							
		**EP**	**WA**	**IP**	**MD**	**RS**	**WP**		
**Species Sampled**	*Anisotremus*	5	3					8	8
	*Conodon*	1	1					2	3
	*Genyatremus*	2	1					3	3
	*Haemulon*	6	13					20	23
	*Haemulopsis*	4						4	4
	*Isacia*	1						1	1
	*Microlepidotus*	2						2	2
	*Orthopristis*	2	2					4	7
	*Pomadasys*	3	2		1	1	2	8	36
	*Xenichthys*	1						1	3
	*Xenistius*	1						1	1
	*Diagramma*			1			1	2	5
	*Plectorhinchus*			7			4	8	35
**Total sampled**		**28**	**22**	**8**	**1**	**1**	**4**	**64**	
**NW Lacking species**	*Boridia*		1						
	Conodon *	1							
	*Haemulon*		3						
	*Orthopristis*	3							
	*Pomadasys*	3							
	Xenichthys *	2							
	*Xenocys*	1							
**Unsampled**		10	4						
**TOTAL**		**38**	**27**						
**% sampled**		**74**	**86**						

Species sampled by ocean basin. EP: eastern Pacific; WA: western Atlantic; IP: Indo–Pacific; MD: Mediterranean; RS: Red Sea; WP: western Pacific. * Doubtful validity of certain species.

Muscle or fin tissue was preserved in ethanol for standard DNA extraction. For most of the Haemulidae species, one or two voucher specimens were kept at CICIMAR-IPN fish collection. Additional specimens for which tissue sample was obtained are associated with their respective photographs and are available upon request.

### DNA extraction, PCR amplification, and sequencing

DNA was extracted following a standard chloroform protocol [55]. PCR amplifications for all species were done for three mitochondrial genes, cytochrome b (CYB), cytochrome oxidase I (COI), and the 16SrRNA, along with two nuclear loci, the first intron of ribosomal protein S7 (S7) and the protein coding recombination-activating gene 2 (RAG2). DNA sequences of CYB, COI, S7 and RAG2 have already been used effectively on grunts [24,31,37]. Primers used for amplification and sequencing are listed in Additional file 2: Table S2. Amplifications were performed in 13 μl reactions containing 0.5 μl of DNA, 0.625 μl of each primer (forward-reverse) and 11.25 μl of Thermo scientific 1.1x PCR master mix (2.5 mM MgCl_2_). After an initial denaturation of 1 to 3 min, 30–35 cycles at 94°C for 45 s, followed by 45 s at an annealing temperature of 52–56°C, and 60 s at 72°C with a final extension of 3 min at 72°C were conducted. Sequencing was performed in one direction with the primers used in the PCR amplification on an ABI 3100 automated sequencer (Applied Biosystems, Foster City, CA) at University of California Berkeley. The putative nature of each sequence was confirmed by BLASTN search. In the case of the nuclear markers, heterozygous individuals were scored using IUPAC ambiguities code.

### Saturation analysis

Xia’s method implemented in Dambe [56] was used to explore saturation within Haemulidae. Sequence divergence is expected to be neither too conservative nor too diverged as to experience substantial substitution saturation; this saturation decreases phylogenetic information [57].

### Phylogenetic analysis

Sequences were trimmed and aligned using the MAFFT [58] routine implemented in Geneious 5.0 (Biomatters). Analyses were performed independently on each gene and on a concatenated matrix in which different set of partitions (by gene, mitochondrial-nuclear, secondary structure for rRNA, proteing coding genes by codon position) and no partitions scenarios were explored. jModeltest 0.1.1 [59] was used to determine the substitution model that best fit the data based on the corrected Aikake Information Criterion.

Two data sets were assembled. The first included only the mitochondrial genes and was used to test the monophyletic status of the family Haemulidae and to explore broader familial relationships, by including a representative of all haemulid genera collected in addition to one or more species from members of different percomorph families, presumed to have some affinity to Haemulidae. The second data set included the combined-matrix of mtDNA, and nucDNA for all sampled haemulids and was used to explore relationships among them.

The phylogenetic analyses exploring familial relationships were rooted using *Beryx splendens*. Beryciforms have been recovered consistently as the sister-group to Percomorpha [16,22,39]. The analyses that dealt with relationships within the haemulins were rooted using two representatives of the *plectorhynchine* subfamily, since they were consistently found to be sisters to the haemuline subfamily. All analyses were done identically for both datasets unless indicated otherwise in the text.

Phylogenetic relationships were assessed using Maximum Likelihood (ML) and Bayesian inference (BI). Maximum Likelihood analyses were performed in GARLI 2.0 [60] and RAxML-GUI 0.93 [61]. The GARLI search was performed in 5 independent runs each automatically terminated after 10000 generations without improving the topology and, specifying the substitution model previously obtained by jModeltest allowing the parameters to be re-estimated during the run. The support was evaluated with 100 bootstrap replicates. The consensus tree from GARLI output, was computed using SumTrees from DendroPy 3.7.0 [62]. RAxML was run 5 times independently with 500 rapid bootstrapping replicates. Majority rule consensus tree was obtained by means of the program Phyutility 2.2 [63].

Bayesian Inference was accessed in MrBayes 3.1 [64] setting priors to fit the evolutionary model suggested by jModeltest but allowing the parameters to be recalculated during the run. Four Markov chains were used to sample the probability space in two simultaneous but completely independent runs starting from different random trees (default option); the number of generations fluctuated depending on the convergence of chains, a sample frequency every 100 generations was performed. The two runs were combined and 25% of the initial trees and parameters sampled were discarded as the burn-in phase. To evaluate if the run was long enough to allow a good chain mixing and accurately represent the posterior probability distribution of all the parameters, the Effective Sample Size (ESS) statistic was evaluated using the software Tracer 1.5 [65]. ESS greater than 200 suggests that MCMC chains were run long enough to get a valid estimate of the parameters.

Sequences were treated gene independent, mitochondrial versus nuclear partition and a total concatenated matrix. Topologies congruencies were assessed by Shimodaira-Hasegawa, and Log Likelihood ratio tests in PAUP 4.0b10 [66]. To avoid systematic error leading to inconsistent topologies due to long branches, out-groups were removed for this test and models and trees were recalculated.

The Bayesian coalescent multispecies and multilocus method (*BEAST) was also explored, as it has demonstrated to perform better than supermatrix analysis in empirical and theoretical data [45]. This method coestimates simultaneously multiple gene trees embedded in a shared species tree, specifying ancestral relationships (topology), and the times ancestral species separated (divergence times). Likelihood ratio test (LRT) was used to test the null hypothesis that the data evolved under a strict molecular clock as implemented in PAUP. Lognormal uncorrelated relaxed clocks were used as rate prior for each gene under this study. All three mitochondrial genes were linked as a single partition and the two nuclear genes left independent. Mixing and convergence of chains was evaluated by means of the Effective Sample Size (ESS) using the software Tracer 1.5 [66].

### Time calibration trees

To test if simultaneous isolation existed under putative geminate pairs [4,35] a time relative tree was inferred in BEAST v1.6.1 [67]. Divergence relative times were estimated under the concatenated matrix. Likelihood ratio test (LRT) was used to test the null hypothesis that the data evolved under a strict molecular clock as implemented in PAUP. The substitution models were the same used in Mr Bayes and a fully bifurcating tree obtained from this search was employed as input for BEAST. This tree was previously prepared in TreeEdit [68] in which MrBayes phylogram was transformed into a chronogram using nonparametric rate smoothing (NPRS) [69]. Yule speciation process was chosen as the BEAST tree prior and the molecular clock model was estimated under a relaxed uncorrelated lognormal distribution [70]. Chain lengths were set to 10 millions of generations with parameters sampled every 1000 (BEAST default). Convergence statistics were monitored by effective samples sizes (ESS), in Tracer v1.5. TreeAnnotator v1.6.1 [71], was used to obtain Maximum clade credibility tree from the 10,000 trees after discarding the first 25% as burn-in.

### Ancestral habitat analysis

Two character states for habitat usage were broadly identified based on published data [8]‐[12,27,43,72-79], for all 54 species included in Haemulinae data set. “Hard bottom” species are those that can be commonly found over coral and/or rocky reefs as opposed to “soft bottom” which are species inhabiting sandy and/or muddy environments.

Ace function of ape [80] (under R v2.13.1) was used to reconstruct ancestral character states using maximum likelihood under the setting (method = "ML" and model = "ARD"). This reconstruction was based on the BEAST maximum clade credibility tree.

### Ancestral area reconstructions

We used data based on present-day distributions [8]‐[12,27,43,72-79] coded as follows: (WA) western Atlantic; (EP) eastern Pacific; and (IP) Indo-Pacific. Three alternative reconstruction methods were used: (i) a Bayesian modified [81]. (ii) A regular dispersal-vicariance analysis [82] (DIVA) both implemented in the computer software Reconstruct Ancestral States in Phylogenies (RASP [83,84]) and, (iii) the dispersal-extinction-cladogenesis analysis (DEC) implemented in the computer program LAGRANGE [85,86]. For accounting on phylogeny uncertainty, ancestral area analyses were carried out on 500 random trees selected from the posterior distribution estimated from BEAST, and information on nodes were summarized and plotted as pie charts.

## Competing interests

The authors declare that they have no competing interests.

## Authors’ contributions

JJT, AAP, and GB carried out the sampling. JJT and GB carried out the molecular laboratory work, performed the molecular analysis and drafted the manuscript. JJT performed the ML ancestral habitat reconstruction and ancestral area analyses. JJT conceived the study. GB, AAP, EFB participated in the design. All authors read, reviewed and approved the final manuscript.

## Supplementary Material

Additional file 1 **Table S1**Material examined. This list indicates sampling information: ocean basin (WA: western Atlantic; EP: eastern Pacific; WP: western Pacific; MD: Mediterranean; RS: Red Sea; IP: Indo–Pacific). Countries code follows ISO list 3166. Voucher catalogue numbers and Genbank accession number are listed. For all New World species at least one specimen was kept as reference and deposited in CICIMAR–IPN fish collection. Photographs for all specimens sampled are available upon request. Click here for file

Additional file 2 **Table S2**Primers used for PCR amplifications followed in this study. Click here for file
