# Supplementary material for: Molecular phylogeny of grunts (Teleostei, Haemulidae), with an emphasis on the ecology, evolution, and speciation history of New World species
Source: BMC Evol Biol. 2012 Apr 26;12:57. doi: 10.1186/1471-2148-12-57 (PMC3472276; doi:10.1186/1471-2148-12-57)
Supplement: Additional file 2 — Table S2Primers used for PCR amplifications followed in this study. [file 1471-2148-12-57-S2.doc]

Supplementary table 2.

| **Gene** | **Primer name** | **Sequence 5' - 3'** | **Reference** |
| --- | --- | --- | --- |
| **16S** | 16SAR | CGC CTG TTT ATC AAA AAC AT | Palumbi 1996 |
|  | 16SBR | CCG GTC TGA ACT CAG ATC ACG | Palumbi 1996 |
|  |  |  |  |
| **COI** | CO1_VF2T1 | TCA ACC AAC CAC AAA GAC ATT GGC AC | Ward et al 2005 |
|  | CO1_VR1dT1 | TAG ACT TCT GGG TGG CCR AAR AAY CA | Ivanova et al 2006 |
|  |  |  |  |
| **CytB** | Gludgl | TGA CTT GAA RAA CCA YCG TTG | Palumbi et al 1991 |
|  | CB3H | GAA TGA TAY TTC CTA TTT GCC | Palumbi et al 1991 |
|  | Hae_Gludgl | GAY GCA YTA RTY GAY CTY CCR GC | This study |
|  | Hae_CB3H | GGG TCY CCI ARI ARR TTI GG | This study |
|  |  |  |  |
| **RAG2** | RAG2-F1 | GAG GGC CAT CTC CTT CTC CAA | Lovejoy 1999 |
|  | RAG2-R3 | GAT GGC CTT CCC TCT GTG GGT AC | Lovejoy 1999 |
|  | RAG2_HaeFw | CTY GAY CCY TAT GAT GGG C | This study |
|  | RAG2_HaeRv | TCT GTG GGT ACA CCA AGT A | This study |
|  |  |  |  |
| **S7** | S7RPEX1F | TGG CCT CTT CCT TGG CCG TC | Chow and Hazama 1998 |
|  | S7RPEX2R | AAC TCG TCT GGC TTT TCG CC | Chow and Hazama 1998 |
